# Water and hydrophobic gates in ion channels and nanopores

**DOI:** 10.1039/c8fd00013a

**Published:** 2018-04-16

**Authors:** Shanlin Rao, Charlotte I. Lynch, Gianni Klesse, Georgia E. Oakley, Phillip J. Stansfeld, Stephen J. Tucker, Mark S. P. Sansom

**Affiliations:** a Department of Biochemistry , University of Oxford , UK . Email: mark.sansom@bioch.ox.ac.uk; b Clarendon Laboratory , Department of Physics , University of Oxford , UK

## Abstract

Ion channel proteins form nanopores in biological membranes which allow the passage of ions and water molecules. Hydrophobic constrictions in such pores can form gates, *i.e.* energetic barriers to water and ion permeation. Molecular dynamics simulations of water in ion channels may be used to assess whether a hydrophobic gate is closed (*i.e.* impermeable to ions) or open. If there is an energetic barrier to water permeation then it is likely that a gate will also be impermeable to ions. Simulations of water behaviour have been used to probe hydrophobic gates in two recently reported ion channel structures: BEST1 and TMEM175. In each of these channels a narrow region is formed by three consecutive rings of hydrophobic sidechains and in both cases such analysis demonstrates that the crystal structures correspond to a closed state of the channel. *In silico* mutations of BEST1 have also been used to explore the effect of changes in the hydrophobicity of the gating constriction, demonstrating that substitution of hydrophobic sidechains with more polar sidechains results in an open gate which allows water permeation. A possible open state of the TMEM175 channel was modelled by the *in silico* expansion of the hydrophobic gate resulting in the wetting of the pore and free permeation of potassium ions through the channel. Finally, a preliminary study suggests that a hydrophobic gate motif can be transplanted *in silico* from the BEST1 channel into a simple β-barrel pore template. Overall, these results suggest that simulations of the behaviour of water in hydrophobic gates can reveal important design principles for the engineering of gates in novel biomimetic nanopores.

## Abstract 2

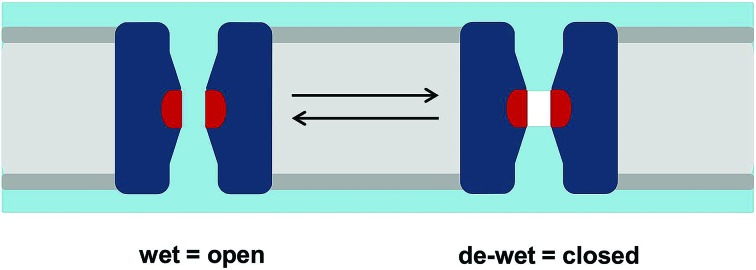
Simulations of water behaviour have been used to probe hydrophobic gates in BEST1 and TMEM175, which can reveal important design principles for the engineering of gates in novel biomimetic nanopores.

## Introduction

Membranes play a key role in the organization of cells. They separate the contents of a cell from the surrounding environment and define individual compartments within cells. Membranes are formed by lipid bilayers, which present hydrophobic barriers that are largely impermeable to water, ions and other solutes. To enable molecules to cross membranes in a regulated fashion, a number of different classes of membrane proteins have evolved. Amongst the simplest of such proteins (in terms of transport mechanism) are ion channels and pore proteins. These are integral membrane proteins which contain a central pore through which selected ions and/or water molecules may flow at near diffusion-limited rates, thus enabling them to cross cell membranes. Ion channels and pore proteins are found in membranes from bacteria, animals and plants, and have a range of functions in the physiology of these cells.

There has been considerable progress in research into the structural biology of ion channels in recent years; over 400 structures of ion channel proteins have been determined to date (http://memprotmd.bioch.ox.ac.uk/_ref/mpm/ion_channels/). Furthermore, the rate of structure determination is rapidly increasing with advances in cryo-electron microscopy.^1^ However, despite these advances, the relationship between a particular molecular structure of an ion channel and its physiological function is not always clear. Computational methods offer the possibility of the rapid and objective structural annotation of new structures of ion channels. Thus, such methods have the potential to complement the functional characterisation using electrophysiological recordings of ionic currents flowing through channels.

One of the most commonly calculated properties of an ion channel structure is the radius profile of the pore.^2^ A display of these physical dimensions provides a primary indicator of the size of ions that may be accommodated within a transmembrane pore and enables one to identify any constrictions or bottlenecks along the length of the pore. Pore radius profiles, and the associated displays of the pore-lining surface, are therefore frequently used to infer the functional state of new channel structures. In particular, if a constriction in a pore is observed, it may correspond to a channel gate controlling whether the channel is open or closed to ion (and water) permeation.

**Fig. 1:**
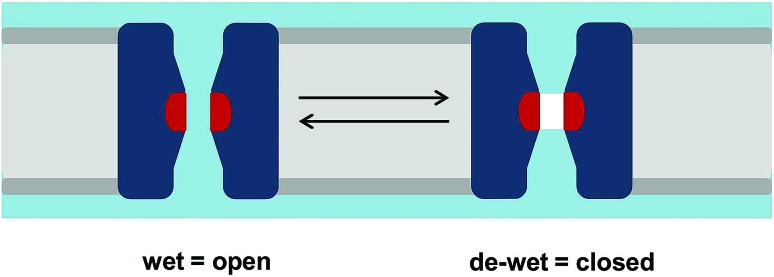
Hydrophobic gating. A schematic of the hydrophobic gating of an ion channel showing how the de-wetting of a hydrophobic constriction (shown in red) can lead to functional closure. The lipid bilayer is shown in grey, the channel in dark blue, and water in pale blue. The vapour (*i.e.* empty volume) in the de-wetted state is shown in white.

The functional consequences of a hydrophobic constriction in a channel may be probed *via* molecular dynamics (MD) simulations. The level of complexity of such simulations may range from extensive characterisation of the free energy landscapes of ion permeation or of conformational transitions associated with gating to simpler potentially high-throughput simulations of the behaviour of water within pores. Some years ago, simulations of simplified models, nanopores and channels were used to develop the concept of hydrophobic gating.^3^–^5^ In essence, this proposes that a sufficiently narrow (typically with a radius of *ca.* 0.4 nm or less) hydrophobic region in a transmembrane pore will spontaneously de-wet to form a nanoscale region that is devoid of water molecules (*i.e.* corresponding to a vapour state that is empty of water molecules on the nanoscale;^6^Fig. 1). In a number of theoretical studies such a region has been shown to form an effective energetic barrier to ion permeation, thus providing a closed state of a channel without requiring full occlusion of the pore. This has been variously referred to as a hydrophobic gate,^3^,^7^ a vapour lock,^8^ or a gate based on nanoscale bubble formation (*i.e.* capillary evaporation).^9^

For ion channels, the concept of hydrophobic gating was first used to explain the nature of the gate in the closed state in members of the Cys-loop family of ligand-gated ion channels such as the nicotinic acetylcholine receptor^10^ and its bacterial homologues (*e.g.* GLIC).^7^ More recently, experimental evidence has emerged for the presence of hydrophobic gates and barriers within other ion channels, such as the TWIK-1 K2P channel,^11^ as well as in synthetic nanopores.^12^ Given the increasing availability of high-resolution structures for many channels and pores, MD simulations can be used to test for hydrophobic gating, suggesting that such computational tools may be used to aid the functional interpretation of novel ion channel structures.^13^

Alongside simulation studies of hydrophobic gating in ion channels, there has been considerable interest more generally in biomimetic ‘nanopores’ in membranes.^14^ Synthetic, biomimetic nanopores have a range of possible applications^15^ including as biosensors,^16^ in water desalination,^17^ and in DNA sequencing.^18^ Nanopores may be formed from a range of materials, both biological (*e.g.* proteins,^19^ peptides^20^ and DNA^21^) and non-biological/synthetic (*e.g.* carbon nanotubes^22^ and track etched nanopores^12^). For such systems to be fully exploited, nanopores need to be gated, *i.e.* capable of controllable switching between functionally closed (impermeable) and open (permeable) states.

Our current understanding of water in nanopores has developed from computational studies of highly simplified water and pore models. Following early work on, for example, the perturbed diffusive and dielectric behaviour of water in nanopore-like confinement,^23^ several studies have demonstrated wetting/de-wetting in simplified models of hydrophobic nanopores^3^,^4^,^24^–^26^ and in carbon nanotubes.^27^,^28^ The concept of hydrophobic gating in nanopores has received direct experimental support from studies of track etched nanopores.^12^

Based on our current understanding of hydrophobic gating we have performed proof-of-principle studies for this as an element of the biomimetic design of novel nanopores based on a protein template.^29^ The designed protein nanopores were shown to undergo electrowetting,^30^ thus providing a mechanism for voltage-regulated gating. Electrowetting is observed in a number of nanosystems: here it enables a hydrophobic gate to be ‘opened’ by the application of a transmembrane voltage, which, if sufficiently large, leads to the wetting of the pore.^25^,^30^ In parallel with computational studies of water in nanopores, there have been advances in our ability to design and implement different biomimetic ‘templates’ for nanopores, *e.g.* by re-engineering pore proteins (*e.g.* OmpG^19^), by designing pore-forming peptides^20^,^31^ and by using DNA origami.^21^,^32^ Alongside advances in the experimental^22^ and computational^33^ characterisation of membrane pores formed by carbon nanotubes, these studies provide a toolkit for the design of hydrophobic gates in a range of nanopores.

Below and in previous studies we show that one can use the presence/absence of water molecules within a pore as a proxy for ion permeability.^3^,^29^ Here we apply simulation analysis to two recently reported ion channel structures: BEST1 (ref. 34) and TMEM175.^35^ These structures are then used in an exploration of the interplay of pore radius and polarity in the (de)wetting/gating of channel pores. A preliminary illustration of the ‘design’ of a hydrophobic gate into a model nanopore is also described.

## Methods

**Fig. 2:**
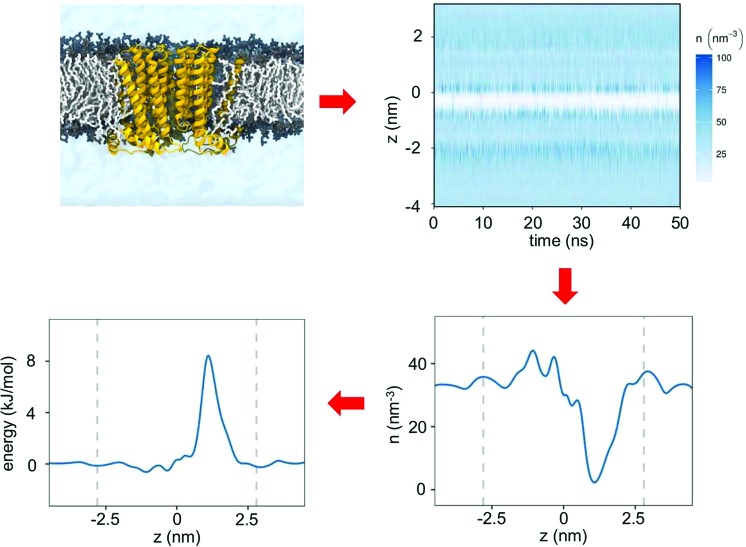
Simulation workflow. MD simulation and analysis of hydrophobic gating, starting with a transmembrane pore domain of an ion channel (yellow) embedded within a lipid bilayer (grey). Analysis of this simulation in terms of the location of water molecules along the pore axis as a function of time yields the number density (in nm^–3^) of water molecules within the pore as a function of the position along the pore axis (*z*). *Via* an inverse Boltzmann transformation this density profile yields a free energy profile for water molecules along the pore axis.

The wetting/de-wetting of a central hydrophobic gate in the transmembrane pore of an ion channel can be examined by tracking the density of water molecules along the pore axis as a function of simulation time (Fig. 2). From this typical example of a hydrophobic gate (*e.g.* in the 5HT_3_R^13^) it can be seen that very few water molecules cross the central hydrophobic region and for most of the simulation the pore is completely empty of water molecules (*i.e.* de-wetted, corresponding to a vapour state). This may be quantified in terms of the number density of water molecules as a function of the position along the pore (*z*) axis. A simple Boltzmann inversion (see below) of the relative density of water within the central hydrophobic region of the pore thus provides an estimate of the energetic barrier height to water permeation in the vicinity of the hydrophobic constriction.

### Simulations

The transmembrane domain from the experimental structure of the channel protein is inserted into a simple phospholipid bilayer *via* a multiscale procedure^36^ resulting in a simulation system comprising the pore domain and is of the order of 300 lipid molecules, 25 000 TIP4P^37^ water molecules, and Na^+^ and Cl^–^ ions of an approximate concentration of 0.15 M.

Simulations were carried out with GROMACS (http://www.gromacs.org) version 4.5.5 (ref. 38 and 39) with the OPLS all atom protein force field with united atom lipids.^40^ Long range electrostatic interactions were treated using the Particle Mesh Ewald method^41^ with a short range cut off of 1 nm, and a Fourier spacing of 0.12 nm. Simulations were performed in the NPT ensemble with the temperature maintained at 310 K with a v-rescale thermostat^42^ and a coupling constant *τ*_T_ = 0.1 ps. Pressure was maintained semi-isotropically using the Parrinello–Rahman algorithm^43^ at 1 bar coupled using *τ*_P_ = 1 ps. The time step for integration was 2 fs with bonds constrained using the LINCS algorithm.^44^ Protein backbones were restrained to their experimentally determined conformations with a force constant of 1000 kJ mol^–1^ nm^–2^.

Simulations were analysed with GROMACS routines, MDAnalysis,^45^ and locally written code. Molecular graphic images were produced with VMD^46^ and PyMOL (; http://www.pymol.org).

### Free Energy Profiles

The distribution of water molecules from the analysis of an equilibrium simulation was used to calculate the free energy of a water molecule as a function of its position in the pore by employing the Boltzmann relation between free energy at a given location and the probability of being in that location:*P*(*z*) = (1/*Z*)exp(–*E*(*z*)/*kT*)where *P* is the probability, *Z* is the partition function, *E* is the free energy and *T* is the temperature. The above probability is directly proportional to d*n*(*z*), the number of water molecules with coordinates between *z* and *z* + d*z* and hence:d*n*(*z*) = *C* exp(–*E*(*z*)/*kT*)where *C* is a constant whose value we set by requiring the value of the free energy to be zero at the two ends of the pore. Inverting the above relation gives:*E*(*z*) = –*kT* ln(d*n*(*z*)) + *kT* ln *C*.

The free energy obtained in this way was plotted against the distance along a pore axis (*z*).

### Pore expansion protocol

An open state model of TMEM175 (see below) was generated by the expansion of the pore in the vicinity of the hydrophobic constriction. Two van der Waals particles were inserted within the hydrophobic gate. These particles had (final) Lennard-Jones 6–12 potential parameters such that *ε* = *ε*(K^+^) and *σ* = 6*σ*(K^+^). The particles were expanded using the Gromacs free energy code, which is similar to the method used in the Alchembed procedure^47^ (with *α* = 0.1, *a* = 1, *b* = 2, *c* = 6) such that *λ* increased from 0 to 1 over a period of 10 ns.

## Results & discussion

### Hydrophobic gates in recently reported structures

**Fig. 3:**
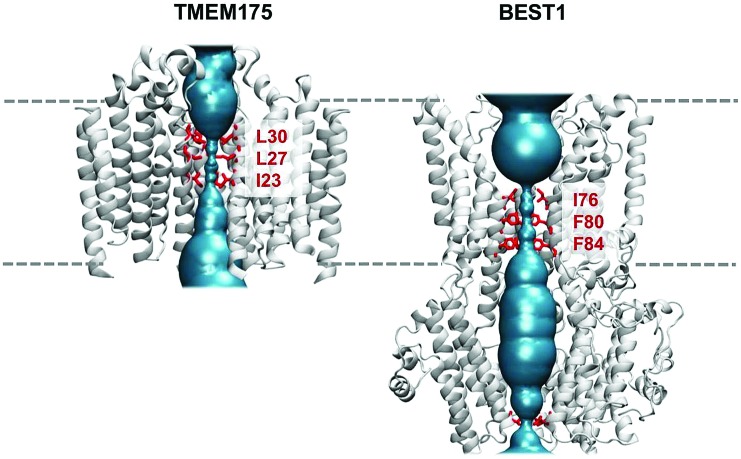
TMEM175 and BEST1 pores. Structures of the TMEM175 (PDB id 5VRE) and BEST1 (PDB id ; 4RDQ) ion channels showing their pore lining surfaces (in cyan) and the rings of hydrophobic sidechains (in red) which line the constricted gate regions in each pore. The broken horizontal lines indicate the approximate location of the lipid bilayer.

Water permeation free energy profiles have been used to identify hydrophobic gates in two ion channels: TMEM175 and BEST1. TMEM175 is a lysosomal potassium channel and a crystal structure (PDB id 5VRE) has been recently determined for a prokaryotic homologue (from Chamaesiphon minutus) of this membrane protein.^35^ This channel has a novel fold, formed by a tetramer of a six-transmembrane-helix (6TM) domain surrounding a central pore (Fig. 3). However, unlike the canonical potassium channel architecture first seen in KcsA^48^ (and subsequently in all other known K^+^-selective channels) TMEM175 does not have a selectivity filter lined by oxygen atoms from carbonyl groups. Rather there is a central constriction within the pore which is lined by three rings of hydrophobic sidechains, namely I23, L27 and L30 (Fig. 3). This hydrophobic constriction in TMEM175 is reminiscent of that seen in another recently reported channel structure, bestrophin (BEST1), which is a Ca^2+^-activated Cl-channel found in the retinal epithelia.^34^ Crystal structures have been determined for BEST1 (PDB id ; 4RDQ) and for its bacterial homologue KpBEST.^49^ Like TMEM175, the BEST1 channel has a hydrophobic constriction of its pore, also lined by three rings of hydrophobic sidechains (I76, F80 and F84). Thus, two novel channel architectures exhibit clear hydrophobic constrictions deep within their transmembrane pores.

In order to probe the functional importance of these hydrophobic constrictions, a number of studies have combined mutagenesis with measurements of ionic currents and/or fluxes.^34^,^35^ However, the picture emerging from these studies remains unclear, thus highlighting some of the difficulties associated with the functional annotation of ion channel structures, even when both mutational and electrophysiological data are available. TMEM175 is selective for K^+^ over Na^+^ ions and the mutation of the isoleucine ring in either the prokaryotic CmTMEM175 or in the homologous human channel leads to a loss of K^+^ selectivity.^35^ This result implicates the hydrophobic constriction in ion selectivity, although the mechanism is far from clear. In contrast, BEST1 is Cl^–^ selective. A ‘triple alanine’ substitution of the three rings of the hydrophobic sidechains (the IFF motif; Fig. 3) which line the hydrophobic constriction results in a wider pore and an increase in overall Cl^–^ flux, but without any change in ionic selectivity. This is consistent with the hydrophobic constriction acting primarily as a gate, rather than a filter.^50^

### MD simulations of water in TMEM175 and BEST1

**Fig. 4:**
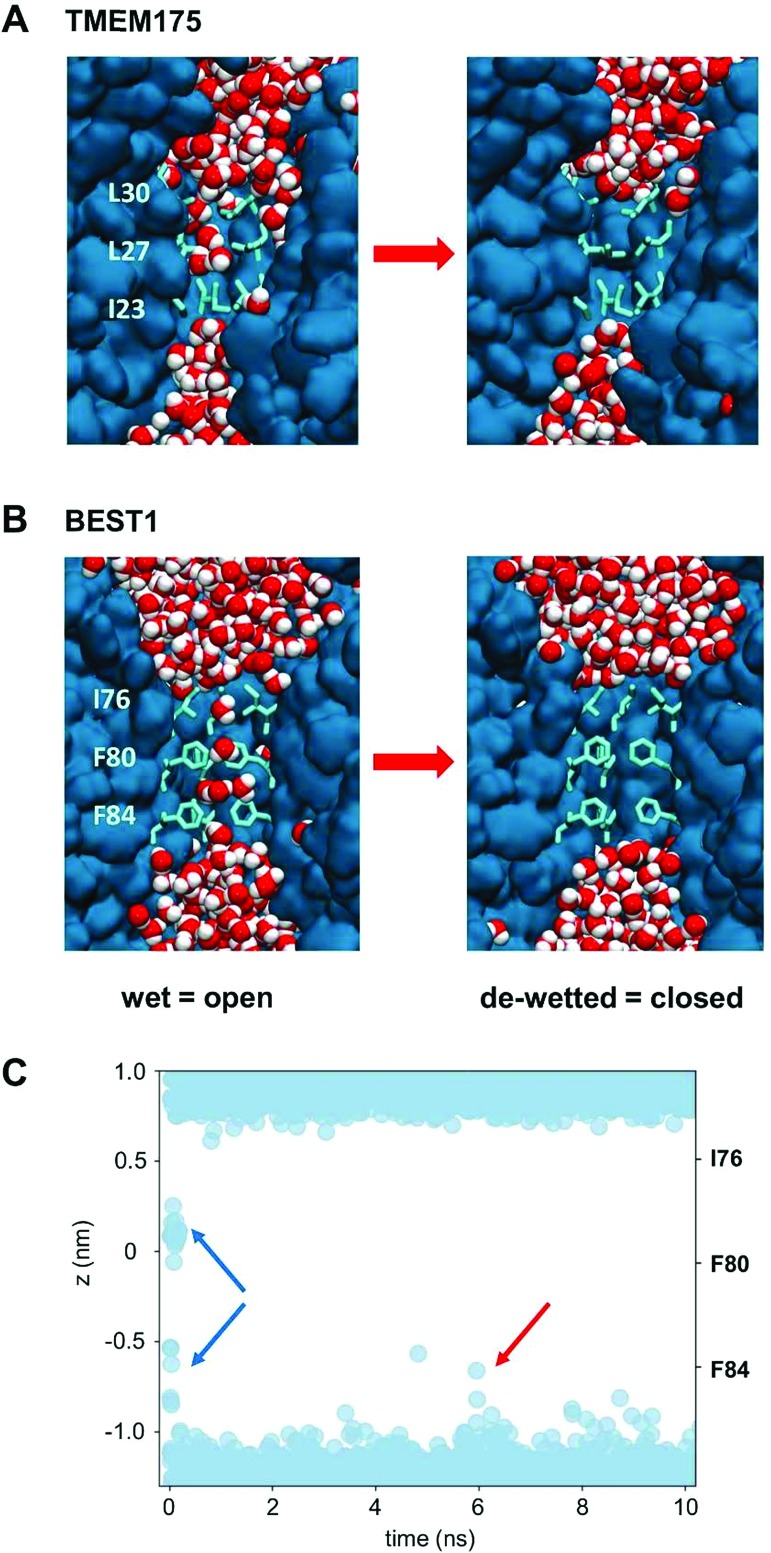
The de-wetting of the pores of TMEM175 and BEST1. (A) The simulation of water molecules (red and white) in the transmembrane pore (blue) of TMEM175. The rings of hydrophobic sidechains lining the pore constriction are shown as pale blue bonds. Water molecules are present in the initial state of the pore (left), which subsequently de-wets (right) within the first few nanoseconds of the simulation. (B) The comparable simulation of water molecules in the transmembrane pore of BEST1. (C) The time course of the de-wetting of the BEST1 pore. The positions of water molecules along the pore axis for the hydrophobic constriction of the BEST1 pore are shown (blue circles), which are taken from the first 10 ns of a MD simulation of the BEST1 channel. It can be seen that there are initially *ca.* 7 water molecules within the constriction (blue arrows). The pore rapidly de-wets, although occasional sporadic forays of isolated water molecules into the constriction occur, *e.g.* as indicated by the red arrow.

**Fig. 5:**
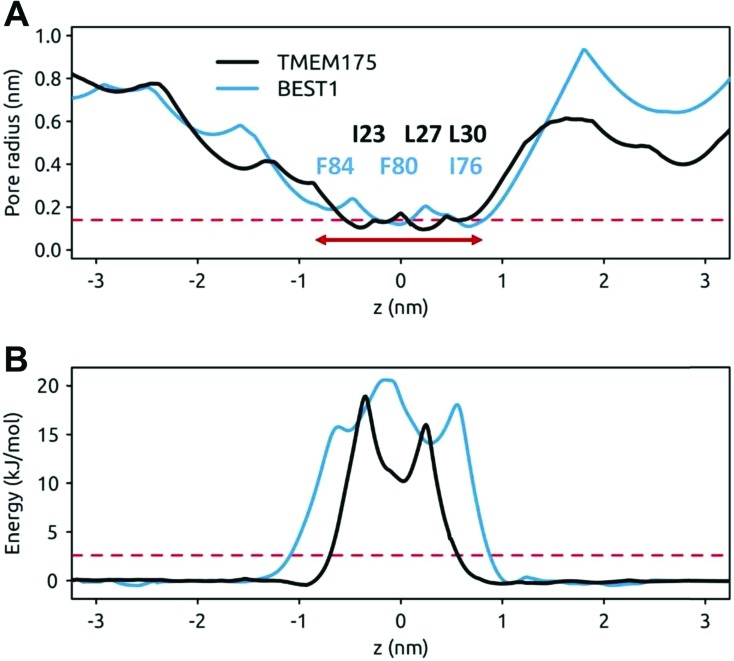
Pore radius and water free energy profiles for TMEM175 and BEST1. (A) Pore radius profiles of the hydrophobic constrictions of TMEM175 (black) and BEST1 (blue line). The broken red line indicates the approximate van der Waals radius of a water molecule (0.15 nm) and the horizontal red arrow indicates the approximate extent of the hydrophobic constrictions. The approximate positions of the hydrophobic pore lining sidechains of the putative gate regions are indicated. (B) Water permeation free energy profiles for the hydrophobic constrictions of TMEM175 (black) and BEST1 (blue). These are derived from 50 ns MD simulations. The broken red line indicates an energy of 1 RT (2.6 kJ mol^–1^).

The question of whether the constrictions present in TMEM175 and the related constriction in BEST1 are hydrophobic gates may be approached *via* molecular dynamics simulations of the behaviour of water within these pores. In both cases the dimensions of the constrictions correspond to a cylinder of radius ∼0.2 nm and length ∼1.5 nm. This should be sufficient to accommodate 5 or 6 water molecules in a single file (a water molecule has a radius of ∼0.15 nm). However, it is clear from the simulations that although the initial solvation of the pores allows several waters to be accommodated (on steric grounds) within the hydrophobic constrictions, after a few nanoseconds of simulation both the TMEM175 and the BEST1 pores de-wet (Fig. 4). The BEST1 pore de-wets early on in the simulations (Fig. 4C) and this is also observed in its prokaryotic homologue KpBEST.^50^ The TMEM175 pore takes several nanoseconds to de-wet, reflecting a need for some degree of mobility of the sidechains in order for the final ‘trapped’ water molecule to escape. Nevertheless, in both cases the pore spontaneously de-wets. This corresponds to an energy barrier for water permeation of 15 to 20 kJ mol^–1^ (Fig. 5). Previous calculations on BEST1 (ref. 50) indicate that this will be associated with an energy barrier to ion permeation of ∼100 kJ mol^–1^. For both TMEM175 and BEST1 the X-ray structure of the channel corresponds to a functionally closed conformation. Thus, by combining these structures with MD simulations of water behaviour, we are able to identify a hydrophobic gate in each of these structures.

### *In silico* mutations of the BEST1 gate

**Fig. 6:**
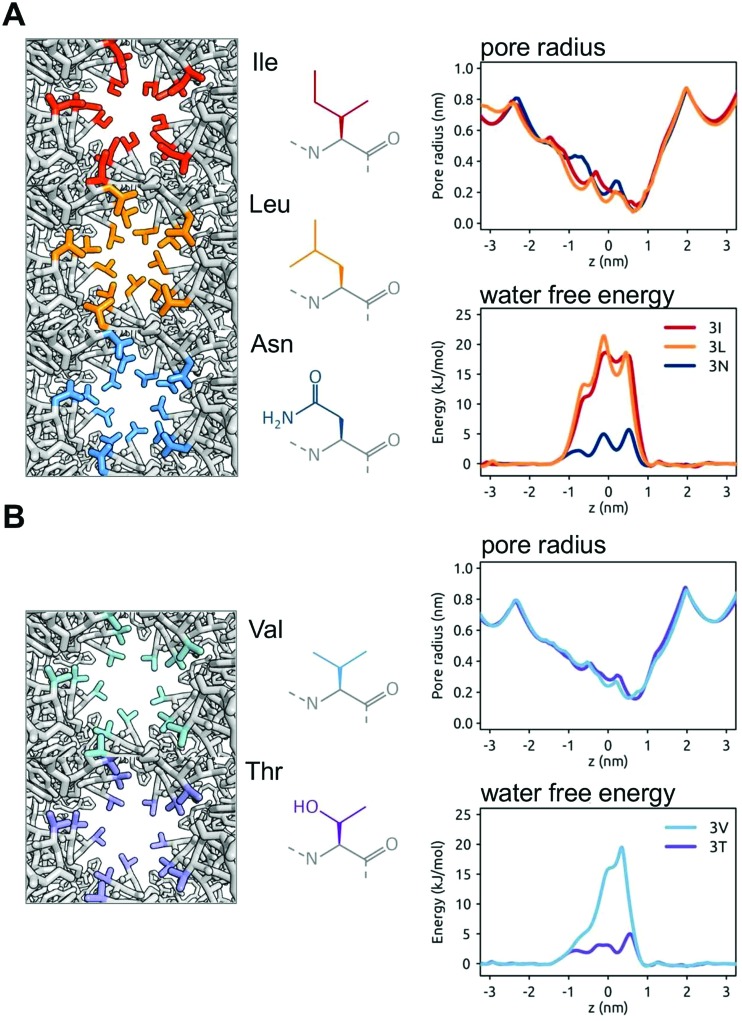
The *in silico* mutation of the hydrophobic gate of BEST1. The wild type IFF gating motif is modified (left hand panels; views down the pore in the region of the gate showing the sidechains of the residues of the gate motif in colour) whilst maintaining the BEST1 channel structure, and the resultant pore radius profiles and water free energy profiles (right hand panels) are shown. (A) The IFF gating motif is replaced by rings of similarly sized sidechains, namely III or LLL (both hydrophobic) or NNN (polar). For III and LLL the free energy barrier to water is similar to that of the wild type BEST1 channel (see Fig. 5B), whereas for NNN the barrier is reduced. (B) The IFF motif is replaced by VVV (hydrophobic) or TTT (polar). Although the pore radius profiles for VVV and TTT are nearly identical, the water free energy profiles are markedly different.

The nature of these hydrophobic gates may be further explored by *in silico* mutations. For BEST1 it has been shown experimentally that the mutation of the IFF motif (*i.e.* the three rings of the I, F and F sidechains lining the gate) to AAA (*i.e.* three rings of smaller, but still hydrophobic alanine sidechains) results in a structure with a wider pore in the gate region (radius of ∼ 3 nm).^34^ Measurement of ionic currents and of chloride ion fluxes demonstrated that the widening of the gate by this triple A mutation greatly increased chloride ion transport rates. Thus, as in earlier simulations of simple model nanopores,^51^ the open *vs.* closed (*i.e.* wet *vs.* dry) nature of a hydrophobic gate depends on the size and the hydrophobicity of the pore. To explore this relationship further we have simulated additional (*in silico*) mutations of the three rings of sidechains lining the hydrophobic gate region of the BEST1 pore. Thus, in addition to the wild type (IFF) and triple A (AAA) mutant pore structures, we have modelled III, LLL, and NNN mutants (Fig. 6A). These three mutants have very similar pore radius profiles, with a minimum radius of <0.15 nm. In the simulations, the two hydrophobic mutants (III and LLL) both yield a free energy barrier to water of ∼20 kJ mol^–1^, which is comparable to that of the wild type BEST1 channel. In contrast, the more polar sidechains of the NNN mutant result in a substantially lower barrier of ∼5 kJ mol^–1^. This has been explored further with two mutants with slightly smaller (and isosteric) sidechains, namely VVV and TTT, both of which yield minimum pore radii of 0.2 nm. For the purely hydrophobic VVV pore lining motif the energetic barrier to water permeation is ∼18 kJ mol^–1^. For the hydrophilic pore lining (TTT) the water permeation barrier drops to ∼3 kJ mol^–1^, *i.e.* a little over RT. Thus, changing the polarity of the sidechain rings results in a clear switch from a closed (VVV) to an open channel (TTT). Preliminary results (not shown) for comparable mutations of TMEM175 suggest a similar pattern is seen.

These results are of interest in the context of recent studies on amino acid side chain hydrophobicity as revealed by simulations of contact angles of water droplets.^52^ Our results for BEST1 gate mutations demonstrate that simulations may be used to obtain a hydrophobicity scale for amino acid sidechains whilst they are lining nanopores. A more global analysis based on simulations of water behaviour in a wide range of ion channel structures will allow us to explore this further (Rao *et al.*, unpublished results).

### Opening a gate by expansion

**Fig. 7:**
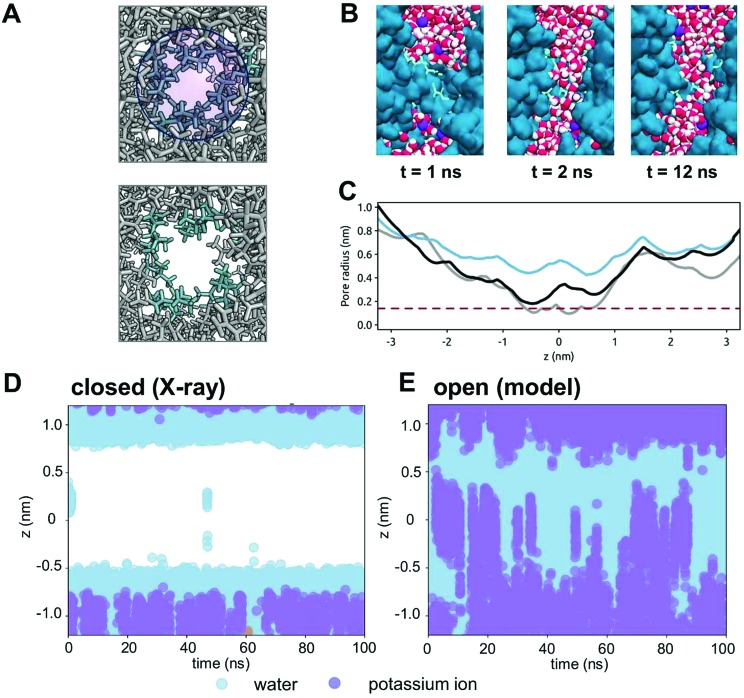
An open state model of the TMEM175 channel. (A) An open state model structure of the TMEM175 channel was developed by the expansion of two van der Waals spheres (blue circle; upper panel) placed within the gating constriction of the pore. The upper panel shows the ILL motif sidechains (cyan) of the crystal structure; the lower panel shows the same region after pore expansion followed by 10 ns relaxation. (B) The simulation of water molecules (red and white) within the transmembrane pore (blue) of the open state model of TMEM175. Initially (*t* = 1 ns) the pore is de-wetted. Shortly after (*t* = 2 ns) the pore is wetted and remains so for the remainder of the simulation. (C) The pore radius profile in the region of the hydrophobic gate for the crystal structure of TMEM175 (grey), for the expanded pore (blue) and for the open state model (black) at the end of a 100 ns unrestrained simulation (see text for details). (D) The position of water molecules (pale blue circles) along the pore axis for the hydrophobic constriction of the TMEM175 pore in the closed state (*i.e.* the X-ray structure). The pore can be seen to be de-wetted for most of the simulation. (E) The positions of water molecules (pale blue circles) and K^+^ ions (purple circles) along the pore axis of the open state model of the TMEM175 pore. The pore can be seen to be wetted throughout the simulation with the entry and exit of K^+^ ions into and out of the pore.

From our analysis of TMEM175 and BEST1, it is evident that a constricted hydrophobic region forms a closed gate in both of these channels. We have used this insight to explore a model of an open state of the TMEM175 pore by driving the expansion of the pore domain in the vicinity of the constriction. Two neutral van der Waals particles were inserted within the hydrophobic gate and smoothly expanded (see Methods for details) over a period of 10 ns (Fig. 7A). This resulted in an increase of the minimum radius of the pore in the hydrophobic gate region from ∼0.1 nm to ∼0.4 nm (Fig. 7C). The inserted particles were then removed and an unrestrained (100 ns duration) simulation performed. This simulation revealed the rapid wetting of the expanded gate region of the pore (Fig. 7B). Over the course of the 100 ns, the minimum radius of the pore relaxed to ∼0.2 nm, but the pore remained wet throughout the simulation. Thus, a small (∼0.1 nm) expansion of the hydrophobic gate of TMEM175 results in a switch from a closed state of the channel, which excludes ions and only allows occasional water molecules to enter the gate (Fig. 7D), to an open state, the expanded gate of which is fully wetted and thus does not present an energetic barrier to the permeation of K^+^ ions (Fig. 7E).

### Transplanting a gate to a model nanopore

Having established the nature of hydrophobic gates in some recently reported ion channel structures, we have then explored whether we can computationally ‘transplant’ a hydrophobic gating motif derived from the BEST1 channel to a simple model of a protein nanopore. We have turned to β-barrel membrane proteins (as found in *e.g.* bacterial outer membranes) to provide a template nanopore in which to transplant the hydrophobic gate. β-Barrel membrane proteins have been widely studied as model nanopores (*e.g.* α-haemolysin^53^ and OmpG^19^) and there has also been progress in the (re)design of β-barrel outer membrane proteins.^54^

**Fig. 8:**
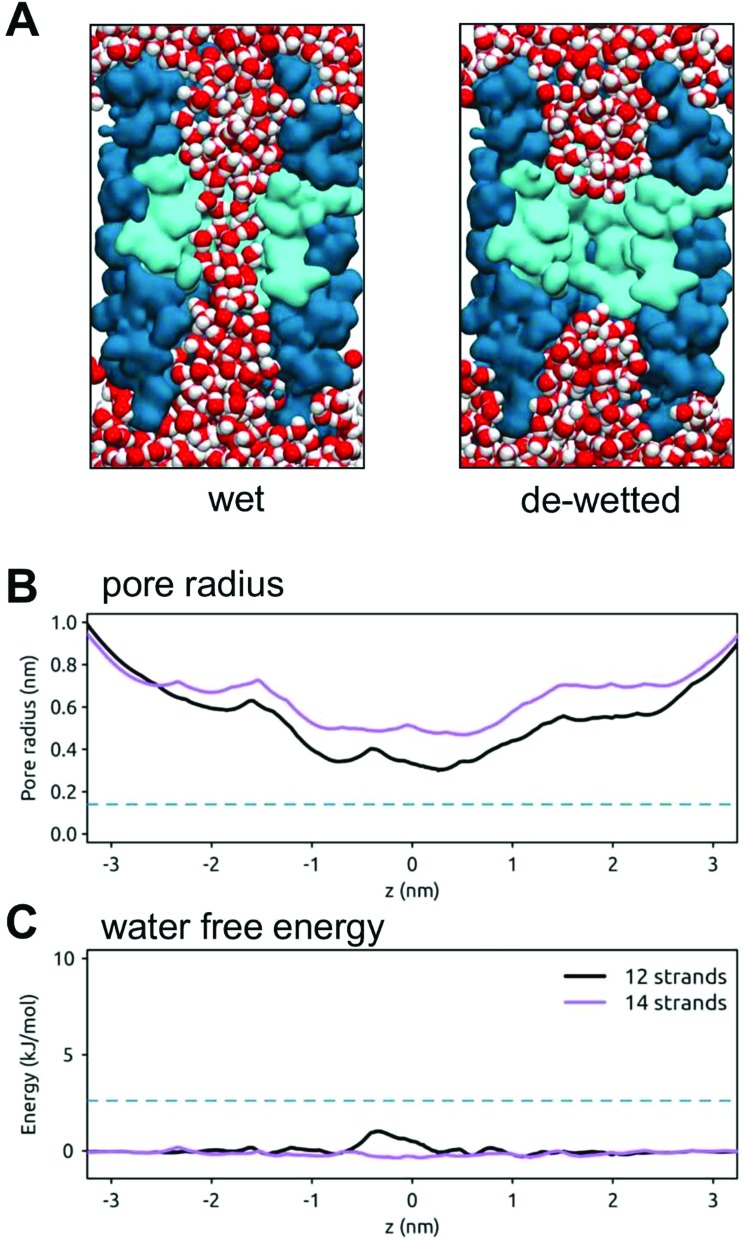
A hydrophobic gate in a model transmembrane nanopore. (A) The wetting and de-wetting of a designed 12-stranded transmembrane β-barrel (shown in cross section in darker blue) with a central hydrophobic constriction (cyan) based on that of the BEST1 channel and formed by consecutive rings of I, F, F and L sidechains. Water molecules are shown in red/white and the lipid molecules of the bilayer have been omitted for clarity. (B) Pore radius profiles for model nanopores based on a 12-stranded (black) and a 14-stranded (purple) β-barrel. The broken horizontal line indicates the radius of a water molecule. (C) Water free energy profiles for model nanopores based on a 12 stranded (black) and 14 stranded (purple) β-barrel. The broken horizontal line indicates an energy of 1 RT (2.6 kJ mol^–1^).

In previous studies we designed a stand-alone hydrophobic gate motif formed by the adjacent rings of leucine residues within a simple β-barrel nanopore template. The resultant pores showed a hydrophobic barrier to water and ion permeation.^29^ We have now extended these studies by ‘transplanting’ a gating motif (IFFL) based on the hydrophobic gate of the BEST1 channel into 12 and 14 stranded template β-barrels (Fig. 8). If transplanted into a 14-stranded barrel the minimum pore radius is ∼0.5 nm and the pore remains open (*i.e.* wet) throughout a 50 ns simulation. If a narrower 12-stranded β-barrel is used as a template, the resultant minimum radius is ∼0.3 nm. This pore stochastically wets and de-wets over the course of a 50 ns simulation. The resultant free energy barrier for water is relatively low (∼2 kJ mol^–1^). Thus, although a gate motif can be transplanted, we need to exert better control over the design of the minimum radius of the hydrophobic gate region.

This also calls for a more global analysis (see above) of free energy barrier heights for water as a function of pore radius and sidechain hydrophobicity in the gate region.

## Conclusions

We have shown that simulations of the behaviour of water within the pore domains of ion channels may be used to analyse possible hydrophobic gates. This is illustrated by two recently reported channel structures of TMEM175 and of BEST1, where simulations clearly reveal the presence of hydrophobic gates and identify the crystal structures as corresponding to the closed states of these channels. Combined with the growth in our knowledge of ion channel structures, this indicates a need for the development of an automated pipeline for running and analysing such simulations for new channel structures as they emerge.^55^ Such a pipeline will also enable a more global analysis of known ion channel structures from the perspective of hydrophobic gating, thus providing a detailed insight into the relationship between pore radius, the hydrophobicity of the pore lining sidechains and the wetting/de-wetting of ion channel proteins.

**Fig. 9:**
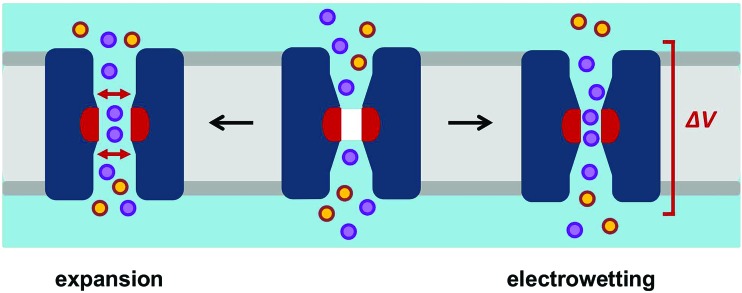
Opening mechanisms for a hydrophobic gate. A schematic diagram of how a hydrophobic gate in a channel/nanopore may be opened, either by expansion or by electrowetting. Once open the constriction within the pore may act as a selectivity filter. The orange and purple circles represent cations and anions respectively.

From our studies to date we can propose two likely opening mechanisms for hydrophobic gates in channels and nanopores (Fig. 9). In biological ion channels it is likely that opening is generally achieved by local pore expansion (as seen in our model of the open state of TMEM175 above), possibly coupled to local changes in conformation such that the pore lining surface becomes less hydrophobic. When designing hydrophobic gates into model nanopores it is possible that electrowetting^30^ may provide a more tractable route to a switchable gate for both water and ion permeable pores, as conformational changes in a relatively rigid β-barrel template may be difficult to engineer. However, before these principles can be fully exploited in the design of novel voltage-activated nanopores, we need more detailed theoretical studies, including *e.g.* the evaluation of the robustness of electrowetting predictions using a range of different water models,^56^ alongside the exploration of different template protein structures for nanopores.^31^

## Conflicts of interest

There are no conflicts to declare.
